# A Guideline-Concordant Chatbot Framework for Structured Colorectal Cancer Screening: Multistage Feasibility Study

**DOI:** 10.2196/93042

**Published:** 2026-07-16

**Authors:** Futao Wu, Xue Li, Yingyi Zeng, Yan Tang, Zhenhua Xiao, Siqi Yang, Kangcheng Wu, Side Liu, Aimin Li

**Affiliations:** 1 Guangdong Provincial Key Laboratory of Gastroenterology, Department of Gastroenterology, Nanfang Hospital, Southern Medical University Guangzhou China; 2 Department of Gastroenterology, Affiliated Qingyuan Hospital, Guangzhou Medical University, Qingyuan People's Hospital Qingyuan China; 3 Department of Gastroenterology, Nanfang Hospital (Zengcheng Branch), Southern Medical University Guangzhou China; 4 Department of Gastroenterology, Zhuhai People's Hospital (Zhuhai Hospital Affiliated with Jinan University) Zhuhai China

**Keywords:** colorectal cancer, cancer screening, clinical decision support, digital health, artificial intelligence, AI

## Abstract

**Background:**

Colorectal cancer (CRC) screening relies on structured risk assessment and guideline-concordant communication, which remain challenging to implement consistently in real-world practice. Digital tools based on large language models (LLMs) may support such workflows, but their feasibility and safety in structured screening contexts have not been well evaluated.

**Objective:**

This study aimed to develop and evaluate a guideline-concordant chatbot framework for structured CRC screening.

**Methods:**

A multistage feasibility study was conducted. In phase 1, baseline performance of contemporary LLMs was assessed using 14 standardized CRC screening questions and validated expert-rated instruments. In phase 2, structured prompt versions were iteratively optimized based on screening guidelines and expert feedback and tested using simulated user scenarios. In phase 3, the optimized chatbot was evaluated in 50 screening-eligible adults to assess feasibility, safety, and guideline concordance.

**Results:**

In phase 1, both LLMs demonstrated satisfactory performance in responding to standardized CRC screening questions, with DISCERN instrument for AI-generated content scores of 12.02 (SD 0.30) for GPT-4o and 13.36 (SD 0.26) for DeepSeek-V3, Global Quality Score scores of 3.96 (SD 0.10) for GPT-4o and 4.39 (SD 0.08) for DeepSeek-V3, Natural Language Assessment Tool for AI scores of 21.41 (SD 0.34) for GPT-4o and 22.73 (SD 0.27) for DeepSeek-V3, and Patient Education Materials Assessment Tool adapted for AI outputs scores of 0.900 (SD 0.016) for GPT-4o and 0.906 (SD 0.015) for DeepSeek-V3. In phase 2, iterative prompt optimization significantly improved all 6 expert-rated dialogue evaluation dimensions (all *P* values <.001). In phase 3, the optimized chatbot successfully collected complete CRC screening risk information and generated guideline-concordant screening recommendations for all participants (N=50). No unsafe or inappropriate outputs were identified.

**Conclusions:**

This study demonstrated the feasibility, preliminary safety, and guideline-concordant performance of a structured chatbot framework for CRC screening communication under the study conditions. Beyond patient education, the proposed framework may support key components of the CRC screening workflow, including risk information collection, risk stratification, and generation of guideline-concordant screening recommendations. Further prospective studies are needed to evaluate the framework’s impact on patient-centered outcomes, screening uptake, and clinical effectiveness.

## Introduction

Colorectal cancer (CRC) is the third most common cancer and the second leading cause of cancer death globally, with about 1.9 million new cases and 935,000 deaths annually [1]. Screening plays a key role in reducing CRC incidence and mortality by enabling early detection and removal of precancerous lesions [2,3]. However, CRC screening uptake remains suboptimal due to low awareness, misconceptions, and psychological barriers such as fear and embarrassment [4,5]. Community-based surveys indicate that real-world adherence to CRC screening ranges from approximately 13% to 55%, even in regions with established screening programs [6]. While the internet has become a major source of CRC-related information, the quality and completeness of online content remain highly variable, potentially impairing public understanding and undermining participation in screening programs [7,8].

Large language models (LLMs) are increasingly applied in health care, including patient education, clinical communication, and decision support [9-12]. Owing to factors such as open-source availability, cost-effectiveness, and suitability for local deployment, LLM-based tools have been rapidly adopted within Chinese health care systems, with reported use in more than 300 hospitals [13]. Despite this growing implementation, evidence supporting their use in CRC screening remains limited. Most existing studies have focused on answering CRC screening–related questions, providing patient education, or disseminating screening guideline information [14-16]. However, CRC screening is a highly structured clinical process that requires standardized information collection, risk assessment, and generation of guideline-concordant screening recommendations. Evidence regarding the application of LLMs within such structured screening workflows remains scarce. In addition, prior studies have raised concerns regarding the quality and reliability of AI-generated medical information [17,18]. Recent evaluations of LLMs in CRC screening have further identified inconsistencies between model-generated recommendations and current screening guidelines [16]. Given that screening eligibility and recommended screening strategies are largely determined by guideline-based criteria, ensuring alignment between LLM outputs and established recommendations is essential for clinical applicability [19].

Although LLMs show promise in CRC screening, their performance in structured screening workflows requiring guideline-concordant recommendations remains insufficiently validated. Accordingly, this study aimed to develop and evaluate an LLM-based chatbot framework for CRC screening. Specifically, we aimed to (1) assess the baseline performance of contemporary LLMs in CRC screening communication; (2) optimize chatbot performance through structured prompt engineering and integration of a localized knowledge framework; and (3) evaluate the feasibility, safety, and guideline concordance of the optimized chatbot in a real-world clinical setting.

## Methods

### Study Design

**Figure 1:**
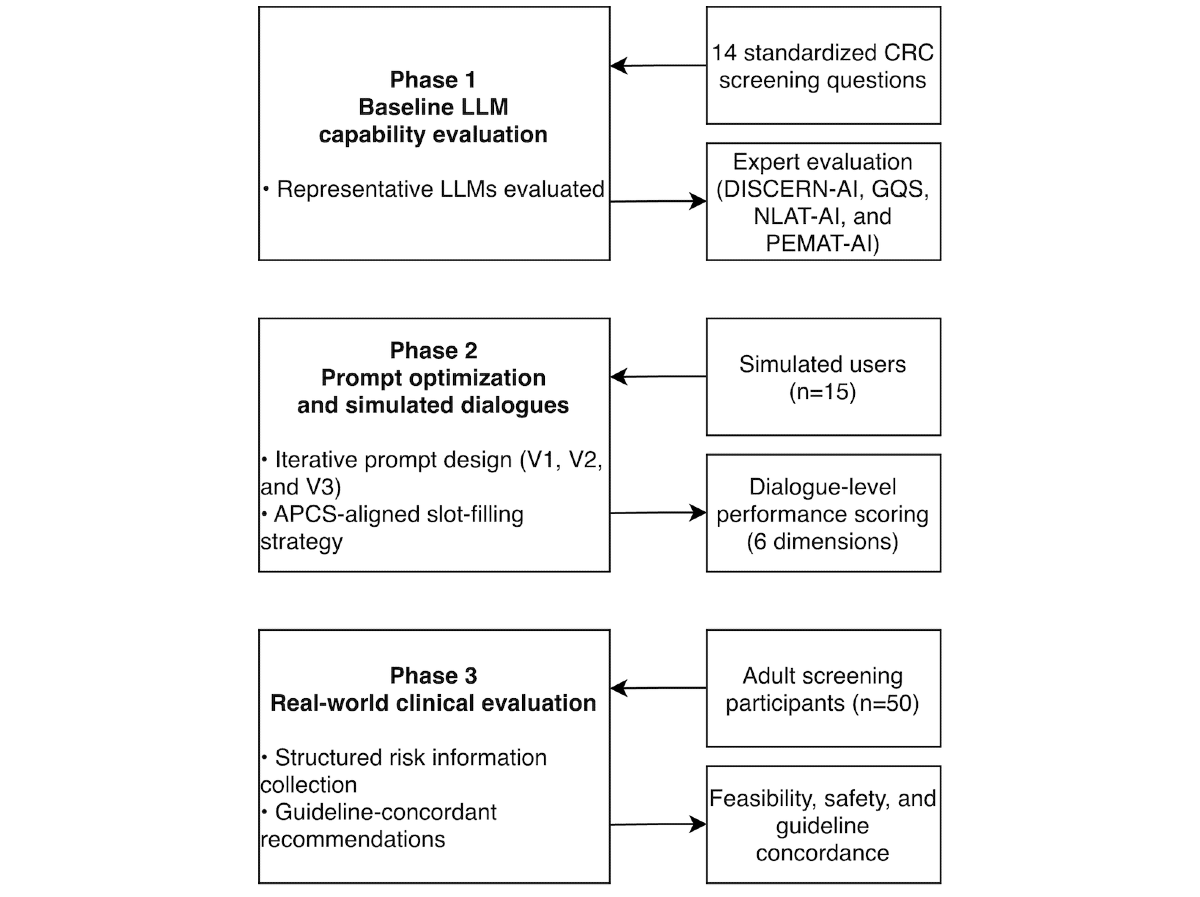
Overview of the multiphase framework for the development and evaluation of an large language module (LLM)–based chatbot for colorectal cancer (CRC) screening. APCS: Asia-Pacific colorectal screening; DISCERN-AI: DISCERN instrument for AI-generated content; GQS: Global Quality Score; NLAT-AI: Natural Language Assessment Tool for AI; PEMAT-AI: Patient Education Materials Assessment Tool adapted for AI outputs.

This study was reported in accordance with the 2025 Transparency in the Reporting of Artificial Intelligence (TITAN) guideline for transparent reporting of AI use in clinical research [20]. A multiphase design was adopted to develop and evaluate an LLM-based chatbot for CRC screening decision support (Figure 1). Phase 1 assessed the baseline performance of 2 representative LLMs in responding to standardized CRC screening–related questions. Phase 2 focused on refining chatbot behavior through iterative prompt optimization and simulated dialogue testing. Phase 3 evaluated the real-world feasibility, safety, and guideline concordance of the optimized chatbot in participants undergoing clinical screening. Across all phases, model outputs and dialogue performance were independently assessed by experienced gastroenterologists using predefined and validated evaluation frameworks.

### Language Model Configuration

Two representative LLMs were included in this study. GPT-4o (OpenAI), a widely used general-purpose LLM that has been extensively evaluated in health care applications, was included as a reference model for baseline comparison. DeepSeek-V3 (DeepSeek), an open-source LLM supporting local deployment, was evaluated in parallel and subsequently selected for chatbot implementation and prompt optimization.

Both models were the latest publicly available versions at the time of model evaluation and prompt development (May 2025; Table S1 in Multimedia Appendix 1). For prompt optimization, the DeepSeek-V3 model was implemented within the Cherry Studio (CherryHQ), which supported prompt design, iterative refinement, and integration of a localized knowledge base derived from established CRC screening guidelines.

### Standardized Questions and Model Evaluation Procedures

A set of 14 standardized CRC screening questions was developed based on a review of the literature, expert input from gastroenterologists, Google Trends data, and key elements of China’s national CRC screening program (Table S2 in Multimedia Appendix 1) [21]. The questions were designed to address major aspects of CRC screening communication, including the necessity of screening, common misunderstandings, risk-related information, and patient concerns.

To minimize potential carryover effects and contextual bias, each interaction was conducted in a newly initialized user session. Conversation history was cleared before each query, and memory-related functions were disabled when applicable to ensure a consistent and clean session state. Each standardized question was submitted as an independent single-turn query, and only the initial model-generated response was retained for analysis. All models were operated using default settings to reflect typical real-world user interactions. Representative responses are provided in Table S3 in Multimedia Appendix 1.

### Simulated User Development

To assess model performance under realistic CRC screening decision-making scenarios, we constructed 15 simulated user profiles based on previously published methodologies and established CRC screening guidelines (Table S4 in Multimedia Appendix 1) [22,23]. User profiles were defined along 3 dimensions: CRC risk level (low, moderate, or high), accuracy of health-related knowledge (accurate vs inaccurate), and screening attitude (neutral vs resistant or anxious). These profiles were systematically generated to cover common combinations encountered in CRC screening practice, resulting in 12 core profiles. In addition, several edge-case profiles incorporating incomplete or extreme information were included to evaluate model robustness. This design enabled coverage of a broad range of information completeness levels and communication challenges commonly encountered in CRC screening interactions.

### Prompt Version Design (V1, V2, and V3)

Three progressively refined prompt versions (V1, V2, and V3) were developed based on prior literature, China’s national CRC screening program, and feedback from preliminary testing (Table 1) [24,25]. Prompt V1 defined the chatbot’s basic role as a CRC screening assistant and was used as a baseline configuration. Prompt V2 introduced a slot-filling strategy to support stepwise collection of key demographic and risk-related information for CRC screening. The collected variables were aligned with the Asia-Pacific colorectal screening (APCS) framework as applied in local screening practice, including age, sex, smoking history, BMI, and family history of CRC. Additional clinically relevant factors, such as prior screening history and alarm symptoms, were also incorporated to support risk stratification and screening decision-making. Prompt V3 further refined response generation by incorporating standardized output formatting, simplified language, and an empathetic communication style to improve clarity, interpretability, and user acceptability. Both prompt V2 and prompt V3 were integrated with a manually curated structured knowledge base derived from national CRC screening guidelines, expert consensus documents, and authoritative patient education materials to support guideline-concordant and culturally adapted responses (Table S5 in Multimedia Appendix 1).

**Table 1:** Iterative prompt design strategy for structured colorectal cancer screening dialogue.

Prompt version	Incremental design purpose	Incremental features
V1: role-based prompt	Establish a defined chatbot role to constrain irrelevant or generic responses.	The model is explicitly designated as a colorectal cancer screening chatbot, focusing exclusively on screening-related information and decision support.
V2: slot-filling prompt (builds on V1)	Enable structured, interactive information gathering.	Several predefined information slots are introduced, and the chatbot requests 1 missing item per turn to support stepwise risk assessment.
V3: personalized output prompt (builds on V2)	Generate personalized, understandable, and actionable advice.	Collected user data are integrated to generate personalized recommendations; output length and structure are constrained to enhance clarity, consistency, and guideline alignment.

To ensure comparability across prompt versions, a standardized user input protocol was applied. All simulated dialogues began with the same initial question (“Do I need to get screened?”), followed by predefined responses to chatbot queries. When the chatbot requested multiple pieces of information in a single turn, only the first requested item was provided according to the predefined simulation protocol. Clarification questions (eg, “Is my risk high?”) were posed if no explicit risk assessment or recommendation was generated, and common follow-up concerns were introduced after recommendations. In total, 45 simulated dialogues (15 users×3 prompt versions) were generated and evaluated. Representative examples are shown in Table S6 in Multimedia Appendix 1.

### Real-World Evaluation in Clinical Participants

On the basis of the optimized prompt configuration developed in the preceding phases, a CRC screening chatbot was implemented and deployed in a clinical setting (Figure S1 in Multimedia Appendix 1). Adult individuals who met the recommended criteria for CRC screening were consecutively recruited from routine screening settings.

Participants interacted with the chatbot to complete structured risk information collection and receive individualized screening recommendations (Figure S2 in Multimedia Appendix 1). All interactions were conducted in a clinical environment, with a trained physician present for observation to ensure procedural safety, without intervening in the chatbot’s responses. Primary outcome measures focused on feasibility and safety. Feasibility was assessed by the successful completion of structured risk information collection and generation of individualized screening recommendations. Safety was assessed through expert review of recommendation appropriateness, guideline concordance, and the occurrence of unsafe or inappropriate outputs, consistent with prior evaluations of LLM-based tools in CRC screening and patient education [19,21]. Outputs were considered unsafe or inappropriate if they contained recommendations inconsistent with established CRC screening guidelines or were judged by expert reviewers to have the potential to adversely affect appropriate screening or follow-up. All chatbot-generated recommendations were independently reviewed by board-certified gastroenterologists using predefined evaluation criteria, and any disagreements were resolved through discussion until consensus was reached.

### Evaluation Metrics and Scoring Instruments

In phase 1, model-generated responses to standardized CRC screening questions were evaluated using 4 validated assessment instruments: the Natural Language Assessment Tool for AI (NLAT-AI), the Patient Education Materials Assessment Tool adapted for AI outputs (PEMAT-AI), the modified DISCERN instrument for AI-generated content (DISCERN-AI), and the Global Quality Score (GQS) [21,26-28]. These instruments assessed key dimensions of screening-related communication, including clinical accuracy, understandability, information reliability, and overall quality. Detailed scoring criteria for each instrument are provided in Tables S7-S10 in Multimedia Appendix 1. In phase 2, chatbot-user dialogues were evaluated using a 6-dimension rubric based on a 5-point Likert scale, adapted from established evaluation frameworks [29,30]. The evaluated dimensions included effectiveness of information gathering strategy, judgment timing appropriateness, recommendation accuracy, personalization and clarity, safety and fairness, and actionability of recommendations. All dialogue outputs were independently rated by 4 board-certified gastroenterologists under single-blind conditions. Detailed definitions and scoring criteria for each dimension are provided in Table S11 in Multimedia Appendix 1.

### Statistical Analysis

Descriptive statistics were used to summarize expert ratings, baseline characteristics, and feasibility-related outcomes. Continuous variables were presented as mean (SD) or median (IQR), as appropriate, while categorical variables were summarized as counts and percentages.

Expert ratings of model-generated responses to standardized CRC screening questions were compared between models using the Wilcoxon signed-rank test. Likert-scale ratings obtained under different prompt configurations were assessed using the Kruskal-Wallis test, followed by Dunn post hoc tests with Bonferroni correction where applicable. Interrater agreement among the 4 board-certified gastroenterologists was assessed using the intraclass correlation coefficient (A,4) based on a 2-way random-effects model with absolute agreement for average measures. The real-world clinical evaluation was analyzed descriptively, with the primary aim of assessing feasibility, safety, and guideline concordance of the chatbot-assisted screening process.

All statistical analyses were performed using R software (version 4.4.1; R Foundation for Statistical Computing). A 2-sided *P* value of <.05 was considered statistically significant. Figures were generated using R software, Microsoft, and BioRender (BioRender Inc).

### Ethical Considerations

Ethics approval for this study was obtained from the medical ethics committee of Nanfang Hospital, Southern Medical University (NFEC-2026-052). All participants provided written informed consent before enrollment. Access to participant data was restricted to authorized study personnel and maintained within a secure, access-controlled environment. Data were deidentified before analysis and publication to protect participant privacy and confidentiality. All study procedures were conducted in accordance with the ethical principles of the Declaration of Helsinki.

## Results

### Model Performance Overview

Across the 4 evaluation instruments, both LLMs demonstrated generally acceptable baseline performance in responding to standardized CRC screening questions (Figure S3 in Multimedia Appendix 1). Mean DISCERN-AI scores were 12.02 (SD 0.30) for GPT-4o and 13.36 (SD 0.26) for DeepSeek-V3, indicating generally adequate information reliability. Mean GQS values were 3.96 (SD 0.10) for GPT-4o and 4.39 (SD 0.08) for DeepSeek-V3, suggesting good overall response quality. For linguistic appropriateness, mean NLAT-AI scores were 21.41 (SD 0.34) for GPT-4o and 22.73 (SD 0.27) for DeepSeek-V3. PEMAT-AI scores were high and comparable between models (GPT-4o: mean 0.900, SD 0.016 vs DeepSeek-V3: mean 0.906, SD 0.015), reflecting similar levels of understandability and structural clarity.

### Prompt Version Comparison

**Figure 2:**
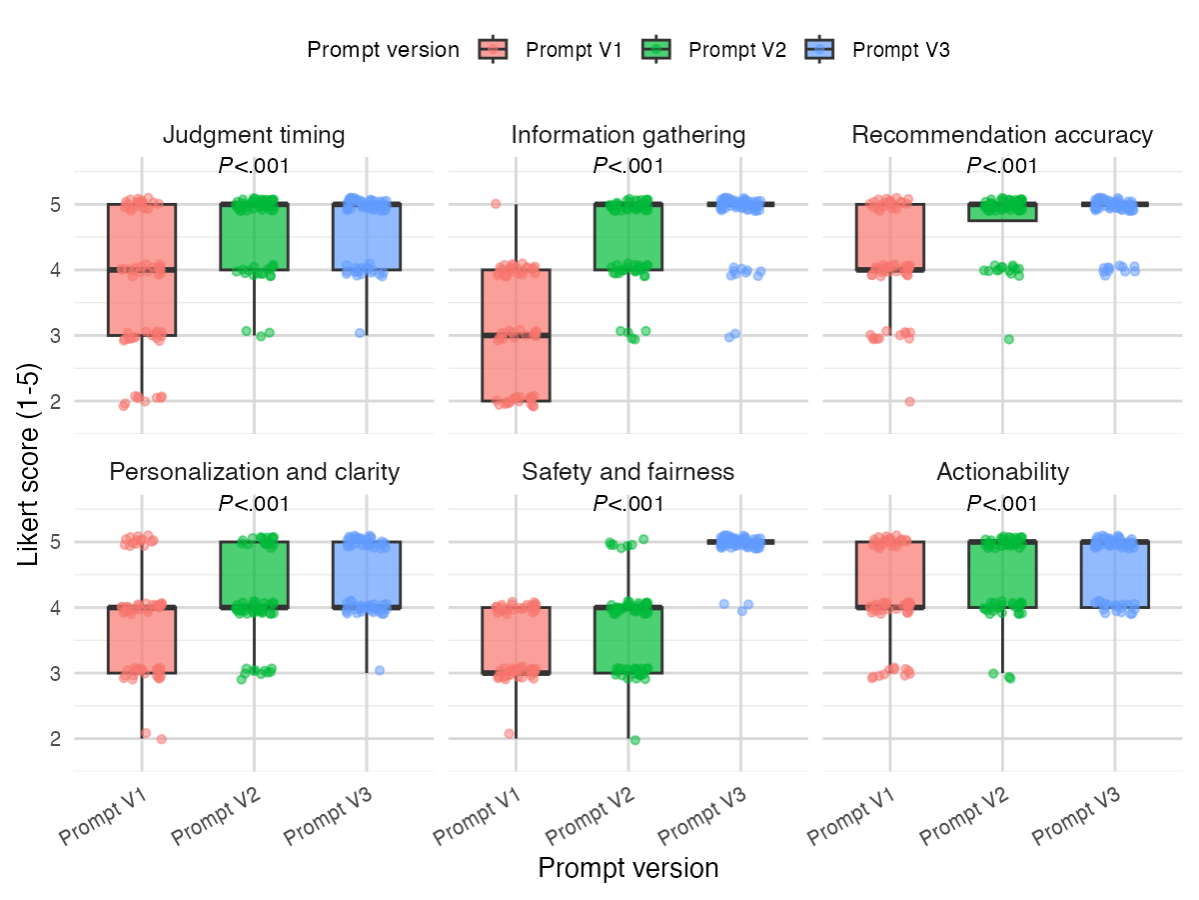
Expert-rated dialogue performance across iterative prompt versions for colorectal cancer screening.

Interrater agreement among the 4 gastroenterologists was acceptable (intraclass correlation coefficient [A,4]=0.685, 95% CI 0.574-0.763; *P*<.001), supporting the reliability of the expert evaluation process. Boxplots illustrate the distribution of expert-rated scores across prompt versions (V1, V2, and V3) for all 6 evaluation dimensions (Figure 2). Median scores increased progressively from V1 to V3 across all dimensions, accompanied by reduced score dispersion in later prompt versions, particularly for information gathering, recommendation accuracy, and safety and fairness. Overall differences among prompt versions were statistically significant across all 6 dimensions (Kruskal-Wallis test, all *P* values <.001). Post hoc analyses using Dunn test with Bonferroni correction showed that prompt V3 scored higher than prompt V1 across all dimensions (adjusted *P*<.05). Compared with prompt V2, prompt V3 demonstrated statistically significant improvements only in personalization and clarity (adjusted *P*=.02) and safety and fairness (adjusted *P*<.001), whereas differences in the remaining dimensions did not reach statistical significance (adjusted *P*>.05).

Radar plots summarize the mean scores of each prompt version across the 6 evaluation dimensions (Figure S4 in Multimedia Appendix 1). From a descriptive perspective, mean scores increased consistently from V1 to V3 across all dimensions, with no dimension exhibiting a decrease in mean performance.

### Real-World Clinical Evaluation

A total of 50 clinical participants were enrolled to assess the real-world feasibility of the optimized CRC screening chatbot (Table 2). Among them, 46% (n=23) were male and 76% (n=38) were aged ≥50 years. Complete CRC screening risk information was collected for all the participants through chatbot-assisted interactions, and all generated screening recommendations were guideline concordant.

APCS-based risk stratification was concordant with the chatbot’s final risk assessment in 92% (46/50) of cases. Expert review confirmed that all chatbot-generated screening recommendations were consistent with current screening guidelines, and no inappropriate or unsafe outputs were identified during the study period.

**Table 2:** Baseline characteristics and feasibility outcomes of real-world chatbot-assisted colorectal cancer (CRC) screening.

		Values (N=50)
**Baseline characteristics**		
	Age (years), mean (SD)	56.8 (8.91)
	Age ≥50 years, n (%)	38 (76)
	Sex (male), n (%)	23 (46)
	BMI (kg/m²), mean (SD)	22.9 (3.85)
	Prior CRC screening, n (%)	15 (30)
	Family history of CRC, n (%)	3 (6)
**Feasibility outcomes of chatbot-assisted CRC screening, n (%)**		
	Complete risk information collected	50 (100)
	Concordance between APCS^a^-based risk stratification and final risk assessment	46 (92)
	Guideline-concordant screening recommendation	50 (100)
	Unsafe or inappropriate outputs	0 (0)

^a^APCS: Asia-Pacific colorectal screening.

## Discussion

### Principal Findings

To our knowledge, this is among the first studies to develop and clinically evaluate a chatbot framework for structured CRC screening. Unlike previous studies that primarily focused on patient education or responses to screening-related questions, our framework was designed to support the entire screening workflow, including risk information collection, risk stratification, and generation of guideline-concordant screening recommendations. To achieve this, we combined iterative prompt optimization with a localized guideline-based knowledge base and evaluated the resulting system in a real-world clinical setting. Together, these findings provide preliminary evidence supporting the feasibility of using LLM-based systems as workflow-oriented screening assistants for guideline-driven CRC screening under the study conditions.

Previous studies have demonstrated that LLMs can provide accurate, complete, and understandable responses to CRC screening–related questions, highlighting their potential value in patient education and health communication [19,31]. In contrast, this study evaluated the potential of LLMs to support structured CRC screening workflows rather than question answering alone. The standardized question set used in our study was designed to cover key components of this workflow, including screening eligibility assessment, risk information collection, risk stratification, screening modality selection, bowel preparation, result interpretation, and follow-up management. Accordingly, our evaluation extended beyond information provision and assessed the potential of LLMs to support structured screening communication and workflow-oriented decision processes. Within this framework, both GPT-4o and DeepSeek-V3 demonstrated satisfactory performance across DISCERN-AI, GQS, NLAT-AI, and PEMAT-AI, suggesting that contemporary LLMs possess not only educational value but also the foundational capabilities required to support structured CRC screening workflows (Figure S5 in Multimedia Appendix 1).

DeepSeek-V3 was selected as the backbone model for CRC screening support because of its open-source availability and suitability for local deployment [32]. These features are particularly relevant for public health applications requiring data privacy, regulatory compliance, and language adaptation [33,34]. Nevertheless, expert reviewers noted occasional use of technical terminology without sufficient explanation, as well as variability in response structure, particularly in follow-up management scenarios. Similar limitations have been reported in previous evaluations of LLMs for CRC screening, where model-generated recommendations did not always align with current screening guidelines and occasionally relied on outdated screening criteria or oversimplified recommendations for high-risk populations [16]. Prior studies have shown that integrating clinical guideline knowledge into LLMs can improve their ability to generate accurate and guideline-concordant responses in medical tasks [24]. Collectively, these observations suggest that translating the general capabilities of LLMs into structured screening support remains challenging and highlight the value of incorporating specialized clinical knowledge into screening-oriented chatbot systems. Our findings further suggest that performance in highly protocol-driven tasks, such as cancer screening, depends not only on the underlying language model but also on how screening workflows and clinical knowledge are structured and integrated into the dialogue system. The progressive improvements observed during iterative prompt refinement, particularly in information completeness and safety, suggest that careful dialogue design can substantially enhance the quality and guideline concordance of screening-related communication. These findings suggest that combining foundation models with domain-specific knowledge and workflow-oriented design may facilitate the development of screening support systems, although further validation is required before clinical implementation and clinical effectiveness can be established. When implemented within a locally deployable, open-source framework, such approaches may facilitate the development of culturally adaptable AI-assisted tools for CRC screening that can be aligned with local screening guidelines, languages, and health care systems. Such characteristics may be particularly relevant for health care settings with stringent requirements for data governance, regulatory compliance, and guideline concordance [35].

In real-world clinical evaluation, the chatbot-generated risk assessment was concordant with APCS-based stratification in most cases, with discrepancies observed in only a small subset of participants. These discrepancies were not attributable to system errors or human intervention. Instead, they reflected the model’s incorporation of clinically relevant contextual information not explicitly captured by the APCS scoring system. Specifically, 2 discordant cases involved participants reporting alarm symptoms, and 1 case involved a participant with a previously positive fecal occult blood test that had not been followed up. In these cases, the chatbot assigned a higher risk assessment than APCS-based stratification and recommended diagnostic colonoscopy. In contrast, 1 discordant case involved a participant classified as high risk according to the APCS who had undergone a normal colonoscopy 1 year earlier. In this case, the chatbot assigned a lower risk assessment and generated a recommendation based on the guideline-recommended surveillance interval. Together, these cases highlight the potential value of incorporating additional clinically relevant information beyond APCS-based stratification for individualized risk assessment. Importantly, all resulting screening recommendations remained consistent with current CRC screening guidelines. This finding highlights an important distinction between population-level risk stratification tools and individualized screening decision support. While APCS is designed to categorize baseline risk, screening recommendations in clinical practice often require integration of additional contextual information beyond the risk score alone. In this study, the chatbot incorporated this information through dialogue-based interactions rather than relying solely on risk score calculation. Although expert review confirmed that all chatbot-generated recommendations remained guideline concordant, the clinical value of incorporating additional contextual information beyond APCS-based risk stratification remains uncertain. Future prospective studies with longitudinal follow-up will be needed to determine whether such recommendation adjustments improve screening outcomes and provide incremental benefit beyond conventional risk stratification approaches. In addition, sustaining the performance of guideline-based chatbot systems in real-world practice will require ongoing alignment between the underlying knowledge base and evolving CRC screening recommendations. Future implementations may incorporate structured expert review and revision of the knowledge base following major guideline updates, along with targeted scenario testing to ensure the continued accuracy and guideline concordance of chatbot-generated recommendations.

This study has several strengths. First, we adopted a stepwise, multistage study design, in which the CRC screening chatbot evaluated in the final phase was systematically developed and optimized based on findings from earlier phases, including baseline model assessment and iterative prompt refinement. Second, by integrating CRC screening guideline knowledge with structured prompt engineering, the chatbot was specifically designed to support a highly protocol-driven screening workflow rather than general health question answering. Third, chatbot outputs were independently evaluated in a single-blind manner by multiple experienced gastroenterologists using validated assessment instruments, supporting the reliability and clinical relevance of the findings. Finally, the use of an open-source and locally deployable LLM may support future adaptation of this framework to other screening and preventive care applications that require guideline-concordant recommendations.

This study has several limitations. First, although real clinical participants were included, the sample size was modest, and the study was designed to assess feasibility and safety rather than comparative clinical effectiveness. Accordingly, direct comparisons with standard clinician counseling, existing screening tools, or usual care were not performed. Second, although chatbot outputs were independently reviewed by experienced gastroenterologists using predefined evaluation criteria, assessment of guideline concordance and clinical appropriateness inevitably involved some degree of subjectivity. Third, patient-centered outcomes, including understanding of screening recommendations, decision confidence, screening uptake, patient behavior, and long-term clinical end points, were not examined. In addition, safety was assessed through expert review of chatbot-generated outputs rather than downstream patient outcomes. Therefore, this study cannot determine whether chatbot use influences delayed care, screening uptake, or other real-world clinical consequences. Fourth, chatbot interactions were conducted in the presence of a trained physician for observation. Although no intervention occurred during chatbot use, this supervised setting may differ from unsupervised real-world deployment and may limit the generalizability of the findings. Finally, as the system was developed and evaluated in a Chinese-language screening context, further validation is required to establish generalizability across other languages and health care systems. Despite these limitations, this work represents an initial step in a sequential research program. Future prospective randomized controlled trials are planned to evaluate the impact of the optimized CRC screening chatbot on patient-centered outcomes, screening uptake, and clinical effectiveness.

### Conclusions

This study developed and evaluated an LLM-based chatbot for CRC screening. By integrating structured prompt engineering with a localized guideline-derived knowledge base, the system demonstrated feasibility, preliminary safety, and guideline-concordant performance under the study conditions. The proposed framework provides a foundation for future research on AI-assisted support in structured screening workflows and may inform the development of similar systems for other clinical tasks requiring standardized information collection and guideline-based recommendations.

## Appendix

Multimedia Appendix 1 Supplementary materials, including technical specifications of the large language models, standardized evaluation questions, prompt versions, simulated user profiles, representative conversations, evaluation instruments, supplementary figures and tables, and additional study results.

## Abbreviations

APCS: Asia-Pacific colorectal screening
CRC: colorectal cancer
DISCERN-AI: DISCERN instrument for AI-generated content
GQS: Global Quality Score
LLM: large language model
NLAT-AI: Natural Language Assessment Tool for AI
PEMAT-AI: Patient Education Materials Assessment Tool adapted for AI outputs
TITAN: Transparency in the Reporting of Artificial Intelligence
