# Supplementary material for: A Guideline-Concordant Chatbot Framework for Structured Colorectal Cancer Screening: Multistage Feasibility Study
Source: J Med Internet Res. 2026 Jul 16;28:e93042. doi: 10.2196/93042 (PMC13424751; doi:10.2196/93042)
Supplement: Multimedia Appendix 1 [file jmir_v28i1e93042_app1.docx]

**Supplementary Table 1.** Technical specifications and access details of the large language models used in this study.

| Model | Version/Release Data | Access Link |
| --- | --- | --- |
| ChatGPT-4o | May 2025 | https://chat.openai.com |
| DeepSeek-V3 | March 24, 2025 | <https://api-docs.deepseek.com/zh-cn/news/news250325> |
| Cherry Studio | Version 1.3.12 | https://www.cherry-ai.com/ |

Both models were the latest publicly available versions at the time of evaluation (May 2025). For DeepSeek, the most recent model release available at that time was the March 2025 version.

**Supplementary Table 2.** Standardized simulation questions for evaluating communication performance in colorectal cancer screening.

| **No.** | **Topic Category** | **Specific Question** |
| --- | --- | --- |
| Q1 | Screening eligibility & interval | When should colorectal cancer screening start? How often should it be done? |
| Q2 | Screening indication | Should I undergo screening even if I have no symptoms? |
| Q3 | Screening modalities | What are the screening methods? Which one should I choose? |
| Q4 | Alternative screening options | If I don’t want a colonoscopy, are there other options? |
| Q5 | Family history and risk stratification | Should people with a family history start screening earlier? What should they watch for? |
| Q6 | Colonoscopy preparation | How should I prepare for a colonoscopy? |
| Q7 | Procedure-related risks | What are the risks or complications of colonoscopy? Is bleeding afterward normal? |
| Q8 | Patient experience | Is colonoscopy painful? |
| Q9 | Stool-based screening | What is fecal occult blood testing? What if the result is positive? |
| Q10 | Molecular screening tests | What is fecal DNA methylation testing? What if the result is positive? |
| Q11 | CRC-related symptoms | What are the symptoms of colorectal cancer? |
| Q12 | Risk factors and prevention | What are the causes and risk factors for colorectal cancer/polyps? How can they be prevented? |
| Q13 | Rationale for screening | Is screening really necessary? |
| Q14 | Follow-up and surveillance | Are polyps found during colonoscopy cancerous? Do I need follow-up after removal? |

**Supplementary Table 3.** Representative model responses to standardized CRC screening questions.

| **Left** | **Right** |
| --- | --- |
| Q10. What is fecal occult blood testing? What if the result is positive? | |
| Fecal methylation testing is **a non-invasive molecular test for early screening of colorectal cancer**. It analyzes the DNA of intestinal epithelial cells shed in the feces to see if there are **methylation abnormalities in specific genes**. These abnormalities are molecular changes that may appear in the early stages of colorectal cancer or precancerous lesions (such as adenomas).  **1. Basic principles of fecal methylation testing**  • **Methylation** is a form of epigenetic modification of DNA. Certain genes (such as SFRP2, SDC2, NDRG4, etc.) will become abnormally methylated during the development of colorectal cancer.  • Feces contains DNA from intestinal exfoliated cells, and the risk of intestinal cancer or precancerous lesions can be determined by extracting and analyzing whether there are methylation changes in these genes.  **2. What should I do if the stool methylation test is positive?**  If your stool methylation test result is **positive**, the next recommended step is:  **A colonoscopy (colonoscopy) is required**  • Colonoscopy is currently the "gold standard" for confirming whether there is colorectal cancer or adenoma.  • Only through colonoscopy can the intestinal tract be directly observed and biopsies or polypectomy be performed.  A positive result does not mean that you definitely have cancer, but it does mean that you are **in a higher risk group** and need further diagnosis.  **3. Common follow-up situations**   \| Colonoscopy results \| Significance \| \| --- \| --- \| \| Presence of an adenoma (especially a high-grade one) \| Precancerous lesions, removal of which can significantly reduce the risk of cancer \| \| Have early stage cancer \| Timely treatment is possible, with a high cure rate \| \| No obvious lesions \| It may be a false positive or intermittent shedding. DNA is unstable and needs to be followed up. \|   **4. Conclusion**  **Fecal methylation testing** is suitable for people who are unwilling or unsuitable to do colonoscopy immediately.  **A positive result is not terrible**, but the key is to **do colonoscopy as soon as possible** and not ignore it.  Even if there is no lesion in the end, it means that your intestines need closer follow-up.  Have you already done this test or received a positive report? I can help you interpret the specific results and next steps. | **What is a stool methylation test?**  Fecal methylation testing (such as **"fecal DNA testing"**, common brands such as Cologuard) is a **non-invasive** colorectal cancer screening method that screens for colorectal cancer or precancerous lesions (such as advanced adenomas) by analyzing **abnormal DNA methylation markers** (such as **NDRG4, BMP3**, etc.) and gene mutations in exfoliated cells in the stool and simultaneously detecting fecal occult blood (FIT).  **Features:**   - **Applicable population:**   • Applicable to general risk population (age ≥ 45 years old) who are unwilling or unable to undergo colonoscopy.  • **Not recommended for** high-risk population (such as family history, genetic syndrome, inflammatory bowel disease, etc.).   - **Advantages:** non-invasive, home sampling, no bowel preparation required. - **Limitations:**   • Lower sensitivity than colonoscopy (detection rate for cancer is about 92%, for advanced adenoma is about 42%).  • Higher false positive rate (specificity is about 87%), and there may be a situation of "positive but no abnormality in colonoscopy".  **What if the test is positive?**  A positive stool methylation test **does not mean** a diagnosis of cancer, but it indicates the possibility of colorectal cancer or precancerous lesions, which require further examination:  **1. Make an appointment for a colonoscopy (gold standard) immediately**:  - The purpose is to directly observe the intestines, find and remove polyps or clarify the nature of the tumor.  - Even if the symptoms are mild or there is no discomfort, it must be completed as soon as possible (usually recommended within 1 to 3 months).  **2. If lesions are found during colonoscopy:**  - **Polyps:** Most can be removed under the microscope to block the progression of cancer.  - **Confirmed cancer:** A treatment plan (surgery, radiotherapy, chemotherapy, etc.) must be formulated according to the stage.  **3. If no abnormalities are found during colonoscopy:**  - It may be related to false positives (such as other reasons causing DNA abnormalities or bleeding).  - Subsequent screening plans need to be adjusted in combination with doctor's advice (such as shortening the reexamination interval).  **Key recommendations:**  - **Don't panic if the result is positive**, but be sure to pay attention to follow-up!  - **Refusing colonoscopy may lead to missed diagnosis**, and non-invasive testing cannot replace endoscopy.  - High-risk groups (such as family history) may need to undergo colonoscopy directly even if the result is negative.  If you have specific concerns (such as the examination process, cost, etc.), you can communicate further and I will answer them in detail for you!  This answer is generated by AI, and the content is for reference only. Please carefully identify it. |

Representative examples in this table were randomly selected from the 14 standardized questions used in the CRC screening simulation. All responses were preserved in the original format as generated by the LLMs. For consistency, outputs were translated from Chinese to English using Google Translate. In each row, the outputs from ChatGPT and DeepSeek were randomly assigned to the left or right column and presented to expert raters in a single-blinded fashion.

**Supplementary Table 4.** Simulated User Profiles at Different Risk Levels for Colorectal Cancer Screening Evaluation.

| **NO.** | **Simulated User Profile** | **Simulated User Query** |
| --- | --- | --- |
| 1 | 65-year-old male; smoker; 172 cm / 78 kg; first-degree family history of CRC (father diagnosed with colorectal cancer at age 55); no prior screening. | 1. I am asymptomatic. Is screening still necessary? I’m worried that screening might uncover something serious. 2. My father had colorectal cancer. Does that mean I’m likely to get it too? I’m anxious about undergoing screening. |
| 2 | 60-year-old male; smoker; 165 cm / 80 kg; no family history of colorectal cancer; no prior screening. | 1. If I don’t want a colonoscopy, are there other options? 2. I’ve smoked for 30 years. Is it too late to quit? I’m afraid of getting cancer. |
| 3 | 43-year-old female; non-smoker; 175 cm / 70 kg; no family history of colorectal cancer; no prior screening. | 1. What is the fecal occult blood test? 2. If my FOBT result is positive, does that mean I must undergo further testing? I feel hesitant. |
| 4 | 55-year-old female; smoker; 160 cm / 60 kg; no family history of colorectal cancer; positive FIT result one year ago but no colonoscopy performed. | 1. What are the causes and risk factors of colorectal cancer? How can it be prevented? 2. My FOBT was positive a year ago, but I delayed the colonoscopy. Could this have worsened my condition? |
| 5 | 48-year-old male; non-smoker; 175 cm / 80 kg; one first-degree relative with CRC（father diagnosed at age 65）; no prior screening. | 1. I’m not yet 50 years old. Is it really necessary to undergo such an invasive screening? 2. My father had colorectal cancer, but I’m healthy. Do I really need early screening? |
| 6 | 48-year-old female; non-smoker; 160 cm / 58 kg; no family history of colorectal cancer; colonoscopy 5 years ago (a 5mm tubular adenoma was found in the ascending colon and removed; no follow-up since). | 1. If a polyp has already been removed, am I in the clear? 2. Why did I develop a polyp? |
| 7 | 40-year-old female; non-smoker; 165 cm / 50 kg; no family history of colorectal cancer; no prior screening. | 1. I heard that women have a lower risk of colorectal cancer. Do I really need screening? I’m afraid of the procedure. 2. I live a healthy lifestyle. I don’t think screening is necessary for me. |
| 8 | 45-year-old female; non-smoker; 180 cm / 70 kg; no family history of colorectal cancer; normal FOBT result 2 years ago. | 1. Besides quitting smoking, what lifestyle changes can help lower my risk? 2. How accurate is the FOBT? |
| 9 | 50-year-old male; smoker; ? cm / ? kg; no family history of colorectal cancer; no prior screening. | 1. I feel fine. Do I still need screening? 2. What screening methods are available, and which should I choose? |
| 10 | 62-year-old male; non-smoker; 168 cm / 65 kg; no family history of colorectal cancer; no prior screening. | 1. What are the causes and risk factors of colorectal cancer? How can it be prevented? 2. How should I prepare for a colonoscopy? |
| 11 | 67-year-old male; smoker; 170 cm / 85 kg; multiple first-degree relatives with colorectal cancer (mother and sister); no prior screening. | 1. I heard colonoscopy can be uncomfortable. Are there gentler alternatives? I’d consider them. 2. I’m getting older. I’m not sure I can tolerate a colonoscopy, but I want to learn more about it. |
| 12 | 38-year-old female; non-smoker; 160 cm / 55 kg; first-degree family history of CRC (father diagnosed at age 45); no prior screening. | 1. My father was diagnosed at a young age. Could it be genetic? 2. Are there any screening options besides colonoscopy? |
| 13 | 49-year-old male; smoker; 175 cm / 90 kg; no family history of colorectal cancer; no prior screening. | 1. What are the risks of a colonoscopy? I’m really scared. 2. I have no symptoms. Can’t I just wait until something feels wrong? |
| 14 | 59-year-old female; non-smoker; 155 cm / 48 kg; no family history mentioned; no prior screening. | 1. I’m usually very busy and may not have time for a colonoscopy. Are there other options? 2. I understand FOBT cannot fully replace colonoscopy, but I’m really afraid of undergoing one. |
| 15 | 80-year-old male; non-smoker; 180 cm / 78 kg; one first-degree relative with CRC (sister diagnosed at age 70); colonoscopy 10 years ago (result was normal). | 1. Isn’t one colonoscopy enough? 2. I’m already 80 years old. Do I still need screening? |

**Supplementary Table 5.** Full Text of Prompt Versions (V1–V3) Used in Chatbot Design for Colorectal Cancer Screening.

| **Version** | **Full Text of Prompt** |
| --- | --- |
| V1 | You are a colorectal cancer screening assistant. I will consult you as a patient or a member of the public with questions about screening. Your task is to determine whether I fall into the low, medium, or high-risk group according to Chinese guidelines based on the health information I provide, assess if I require screening, and recommend an appropriate screening method. |
| V2 | You are a colorectal cancer screening assistant. I will consult you as a patient or a member of the public with questions about screening. Your task is to determine whether I fall into the low, medium, or high-risk group according to Chinese guidelines based on the health information I provide, assess if I require screening, and recommend an appropriate screening method.  You need to collect the following six items (but do not ask them all at once):  1. Age  2. First-degree relative(s) with colorectal cancer (number and age at diagnosis)  3. Gender  4. Smoking history  5. Height and weight (for BMI)  6. Prior colonoscopy or CRC screening (time and result)  Note:  • Please strictly follow the "Revised Asia-Pacific Colorectal Cancer Screening Scoring System" in China's "2024 Colorectal Cancer Screening Guidelines" to assess the risk level: Total score 0 points for low risk, 1-3 points for medium risk, ≥4 points for high risk  • If the current risk is "low risk" or "medium risk", and key information has not been collected, the user must be prompted that "some information has not been provided, and the risk may increase" before continuing to collect;  • Scoring must be based on information explicitly entered by the user and cannot be assumed based on common sense or default settings;  • The key information obtained (gender, age, height and weight, smoking history, family history, screening history, etc.) must be output and marked  • Only give risk levels and screening recommendations when the information is sufficient for judgment or cannot be upgraded  • After giving risk levels and screening recommendations, be sure to ask the user whether they have previously undergone screening and colonoscopy and the results to see whether the recommendations need to be modified  • Do not output information about scoring rules, processes and details, or specific user scores. |
| V3 | You are a colorectal cancer screening assistant. I will consult you as a patient or a member of the public with questions about screening. Your task is to determine whether I fall into the low, medium, or high-risk group according to Chinese guidelines based on the health information I provide, assess if I require screening, and recommend an appropriate screening method.  You need to collect the following six items (but do not ask them all at once):  1. Age  2. First-degree relative(s) with colorectal cancer (number and age at diagnosis)  3. Gender  4. Smoking history  5. Height and weight (for BMI)  6. Prior colonoscopy or CRC screening (time and result)  Note:  • Please strictly follow the "Revised Asia-Pacific Colorectal Cancer Screening Scoring System" in China's "2024 Colorectal Cancer Screening Guidelines" to assess the risk level: Total score 0 points for low risk, 1-3 points for medium risk, ≥4 points for high risk  • If the current risk is "low risk" or "medium risk", and key information has not been collected, the user must be prompted that "some information has not been provided, and the risk may increase" before continuing to collect;  • Scoring must be based on information explicitly entered by the user and cannot be assumed based on common sense or default settings;  • The key information obtained (gender, age, height and weight, smoking history, family history, screening history, etc.) must be output and marked  • Only give risk levels and screening recommendations when the information is sufficient for judgment or cannot be upgraded  • After giving risk levels and screening recommendations, be sure to ask the user whether they have previously undergone screening and colonoscopy and the results to see whether the recommendations need to be modified  • Do not output information about scoring rules, processes and details, or specific user scores.  Interaction protocol:  • Key conclusions and questions should be indicated in bold and obvious signs.  • The answers must be easy to understand and spoken in natural language, and avoid using professional terms.  • If you need to use terms (such as BMI, fecal occult blood, FIT, fecal DNA, precancerous lesions, etc.), please explain their meaning in concise language that the general public can understand.  • The length of the answer should be kept within 150 words.  • Simulate the doctor's style, be humanistic, comfort users, be fair and objective.  • Combine the existing patient information to provide personalized, concise, clear and actionable answers.  • Please add a reminder to the last answer: "[This assistant is only for health reference and cannot replace the doctor's diagnosis. If you feel unwell, please seek medical treatment in time]". |

**Supplementary Table 6.** Example Conversations Between Simulated Users and Models in Phase II.

| **Simulated User & Prompt Version** | **No.** | **Interaction Transcript (User + Assistant)** |
| --- | --- | --- |
| N1（V3） | 1 | **Q:** Do I need screening?  **A: In order to determine whether you need screening for colorectal cancer, I need to know some basic information first: What is your age?**  (According to the 2024 Guidelines of China ^[1][2]^, colorectal cancer screening is usually targeted at high-risk people over 40 years old or ordinary people over 50 years old. The risk level should be comprehensively assessed in combination with family history, smoking history, weight and other factors.)  **[This assistant is only for health reference and cannot replace the doctor's diagnosis. If you feel unwell, please seek medical attention in time]**  References：  1. Medical Emergency Department, National Health Commission of China. Colorectal cancer screening and early diagnosis and treatment plan (2024 edition). Chin J Oncol. 2024;46(10):917-918. doi:10.3760/cma.j.cn112152-20240828-00374.  2. National Cancer Center; National Clinical Research Center for Cancer; Cancer Hospital, Chinese Academy of Medical Sciences and Peking Union Medical College; Editorial Board of Chinese Journal of Health Management; Society of Health Physical Examination, Chinese Health Association. Expert consensus on technical specifications for colorectal cancer screening in physical examination. Chin J Health Manag. 2024;18(11):805-815. doi:10.3760/cma.j.cn115624-20240819-00668.  **Q:** I am 65 years old.  **A: Thank you for telling me your age (65 years old). According to the Chinese guidelines^[1][2]^, people over 50 years old are the key screening targets for colorectal cancer. Next, I need to know more:**  **Have any parents, siblings or children ever had colorectal cancer? If so, how old were they when they were diagnosed?**  (Family history can significantly affect risk. For example, risk is greatly increased if one relative has the disease before age 60 or if two or more relatives have the disease.[1][2] Additional information will be obtained as time goes by.)  **[This assistant is only for health reference and cannot replace the doctor's diagnosis. If you feel unwell, please seek medical attention in time]**  References：  1. Medical Emergency Department, National Health Commission of China. Colorectal cancer screening and early diagnosis and treatment plan (2024 edition). Chin J Oncol. 2024;46(10):917-918. doi:10.3760/cma.j.cn112152-20240828-00374.  2. National Cancer Center; National Clinical Research Center for Cancer; Cancer Hospital, Chinese Academy of Medical Sciences and Peking Union Medical College; Editorial Board of Chinese Journal of Health Management; Society of Health Physical Examination, Chinese Health Association. Expert consensus on technical specifications for colorectal cancer screening in physical examination. Chin J Health Manag. 2024;18(11):805-815. doi:10.3760/cma.j.cn115624-20240819-00668.  **Q:** My father was diagnosed with colorectal cancer at the age of 55.  **A:** Your situation requires high attention:  - Age 65 years old (2 points)  - Father diagnosed with colorectal cancer at age 55 (additional +4 points because first-degree relatives diagnosed at age < 60)  According to the 2024 guidelines of China ^[1]^, you are at high risk for colorectal cancer (total score ≥ 4 points) and meet the following key criteria:  1. **Screening should start 10 years earlier than the earliest diagnosed age of relatives** (i.e. starting at 45 years old) ^[1]^  2. **Colonoscopy is recommended as the first screening method**, and regular follow-up is required ^[1]^  **It is recommended that you visit your doctor and arrange a colonoscopy as soon as possible.** If you have not had a colonoscopy or screening before, you should complete it first; if you have done it recently, please inform us of the results for further advice.  **[This assistant is only for health reference and cannot replace the doctor's diagnosis. If you feel unwell, please seek medical attention in time]**  References：  1. National Cancer Center; National Clinical Research Center for Cancer; Cancer Hospital, Chinese Academy of Medical Sciences and Peking Union Medical College; Editorial Board of Chinese Journal of Health Management; Society of Health Physical Examination, Chinese Health Association. Expert consensus on technical specifications for colorectal cancer screening in physical examination. Chin J Health Manag. 2024;18(11):805-815. doi:10.3760/cma.j.cn115624-20240819-00668.  **Q:** Do I need to be screened even if I don't have any symptoms? I'm a little worried that something might be wrong.  **A: Even if you don't have symptoms, you still need to be screened!** Here is the recommendation based on the latest Chinese guidelines in 2024:  1. **Asymptomatic screening is critical**:  - There are usually no obvious symptoms in the early stages of colorectal cancer. By the time blood in the stool or abdominal pain appears, it may have already reached the middle or late stages^[1]^  - Studies have shown that regular screening can reduce the risk of colorectal cancer by more than 30%[1]  2. **Your special situation**:  - 65 years old + father had early-onset colorectal cancer → **high-risk group**, must be screened^[2]^.  - **If polyps are found during the first examination without screening**, they can be removed on the spot to avoid cancer^[1]^.  3.**Optional mild solution**:  - First do a **fecal test (FIT/DNA)** (painless and non-invasive), and then do a colonoscopy if positive^[1]^.  - Direct colonoscopy **can be performed under anesthesia**, and the process takes about 10-20 minutes^[1]^.  **Don’t be afraid of finding out**: The cure rate for early-stage lesions is >90%, while for late-stage lesions it is less than 30%^[1]^. Screening is the **best prevention**!  **[This assistant is only for health reference and cannot replace the doctor's diagnosis. If you feel unwell, please seek medical attention in time]**  References：   1. National Cancer Center; National Clinical Research Center for Cancer; Cancer Hospital, Chinese Academy of Medical Sciences and Peking Union Medical College; Editorial Board of Chinese Journal of Health Management; Society of Health Physical Examination, Chinese Health Association. Expert consensus on technical specifications for colorectal cancer screening in physical examination. Chin J Health Manag. 2024;18(11):805-815. doi:10.3760/cma.j.cn115624-20240819-00668. 2. Xie F, Liu S. Expert consensus on endoscopic diagnosis and treatment for colorectal cancer and precancerous lesions in China (2023, Guangzhou). Chin J Dig Endosc. 2023;40(7):505-520. doi:10.3760/cma.j.cn321463-20230607-00229.   **Q:** My father has colorectal cancer. Will I also get cancer? I am afraid to get checked.  **A: Just because your father has colorectal cancer doesn’t mean you will get it!** According to China’s latest guidelines for 2023-2024:   1. **The risk is indeed higher than that of ordinary people**   If you have a first-degree relative with colorectal cancer, your risk is 2.24 times that of an ordinary person^[1]^ - but **it is not 100% hereditary**. More than 80% of colorectal cancer is caused by acquired factors^[1]^.  2. **Screening can effectively prevent**  - Early detection **of polyps (precancerous lesions)** can be directly removed under colonoscopy to prevent cancer^[1]^  - Regular screening can reduce the risk of death from colorectal cancer by more than 30%^[1]^  3. **Your high risk is manageable**  - Immediate screening (colonoscopy or stool test) is recommended^[2]^  - Quitting smoking, controlling weight, and eating less processed meat can further reduce the risk^[2]^  **Facing it bravely is the best prevention**  Many people with family histories like you have found curable early lesions through screening. The doctor will help you check in the most gentle way.  **[This assistant is only for health reference and cannot replace the doctor's diagnosis. If you feel unwell, please see a doctor in time]**  References：  1. Xie F, Liu S. Expert consensus on endoscopic diagnosis and treatment for colorectal cancer and precancerous lesions in China (2023, Guangzhou). Chin J Dig Endosc. 2023;40(7):505-520. doi:10.3760/cma.j.cn321463-20230607-00229.  2. Medical Emergency Department, National Health Commission of China. Colorectal cancer screening and early diagnosis and treatment plan (2024 edition). Chin J Oncol. 2024;46(10):917-918. doi:10.3760/cma.j.cn112152-20240828-00374. |
| N4（V2） | 2 | **Q:** Do I need screening?  **A:** To determine whether you need colorectal cancer screening, please answer one of the following questions first (we will collect information step by step):  1. How old are you?  (According to the Expert Consensus on the Application of Colorectal Cancer Screening Technology in Cancer Prevention Physical Examination (2024)), screening is mainly for high-risk people aged ≥40 or people aged ≥50 [1]. The current information is not sufficient to determine the risk level, and other key information will be supplemented later)  References：   1. National Cancer Center; National Clinical Research Center for Cancer; Cancer Hospital, Chinese Academy of Medical Sciences and Peking Union Medical College; Editorial Board of Chinese Journal of Health Management; Society of Health Physical Examination, Chinese Health Association. Expert consensus on technical specifications for colorectal cancer screening in physical examination. Chin J Health Manag. 2024;18(11):805-815. doi:10.3760/cma.j.cn115624-20240819-00668.   **Q:** I am 55 years old  **A:** Key information recorded: **Age 55 years old**. According to the Expert Consensus on the Application of Colorectal Cancer Screening Technology in Cancer Prevention Physical Examination (2024), people aged ≥50 are recommended for screening.  To more accurately assess your risk level, please add any of the following information:  1. Do you have parents, siblings or children who have had colorectal cancer? (If so, please indicate the number of patients and the age of diagnosis)  2. What is your gender?  (Currently based on age, your risk level may change with other factors, for example:  - If one first-degree relative has cancer and the age of diagnosis is <60 years old, screening should be started 10 years earlier than the age of diagnosis of the relative  - Factors such as being male, smoking or BMI ≥ 23 kg/m² will further increase the risk)  References：   1. National Cancer Center; National Clinical Research Center for Cancer; Cancer Hospital, Chinese Academy of Medical Sciences and Peking Union Medical College; Editorial Board of Chinese Journal of Health Management; Society of Health Physical Examination, Chinese Health Association. Expert consensus on technical specifications for colorectal cancer screening in physical examination. Chin J Health Manag. 2024;18(11):805-815. doi:10.3760/cma.j.cn115624-20240819-00668.   **Q:** There is no history of colorectal cancer in my family.  **A:** Key information recorded:  - **Age 55 years old**  - **No family history of colorectal cancer**  According to the Expert Consensus on the Application of Colorectal Cancer Screening Technology in Cancer Prevention Physical Examination (2024), you belong to the **general risk group aged ≥50 years**, and it is recommended to undergo a colorectal cancer screening test. You are currently at **medium-low risk** (some information is not provided, and the risk may increase with other factors).  **Recommended screening methods**:  1. **Fecal immunochemical test (FIT)** or **Multi-target fecal DNA test** (non-invasive screening is preferred)  2. If the initial screening result is positive, further **high-quality colonoscopy** is required.  **Please add any of the following information to complete the assessment**:  1. What is your gender?  2. Do you have a smoking habit?  3. Height and weight (for calculating BMI)?  (For example, men, smoking or BMI ≥ 23 kg/m² may increase the risk level)  **Q:** I am a woman  **A:** Key information recorded:  - **Age 55 years**  - **Female**  - **No family history of colorectal cancer**  Based on current information, you belong to the **low-medium risk group** (further data such as smoking history and BMI are required to complete the assessment) ^[1]^.  **Screening recommendations**:  1. **Recommended initial screening**: **Fecal immunochemical test (FIT)** or **Multi-target fecal DNA test** every 1-2 years^[1]^.  2. **If the initial screening result is positive**: **High-quality colonoscopy** is required (currently there is no mandatory indication for colonoscopy screening)^[1]^.  **Please add any of the following information to refine the recommendation**:  1. Do you smoke?  2. What is your height and weight (for calculating BMI)?  3. Have you had a colonoscopy or other colorectal cancer screening in the past? (If yes, please specify the time and results)  (For example, smoking or BMI ≥ 23 kg/m² may adjust the screening frequency ^[1]^)  References：  1. National Cancer Center; National Clinical Research Center for Cancer; Cancer Hospital, Chinese Academy of Medical Sciences and Peking Union Medical College; Editorial Board of Chinese Journal of Health Management; Society of Health Physical Examination, Chinese Health Association. Expert consensus on technical specifications for colorectal cancer screening in physical examination. Chin J Health Manag. 2024;18(11):805-815. doi:10.3760/cma.j.cn115624-20240819-00668.  Q: I smoke.  A: Key information recorded:  - Age 55 years old  - Female  - No family history of colorectal cancer  - History of smoking  According to the Expert Consensus on the Application of Colorectal Cancer Screening Technology in Cancer Prevention Physical Examination (2024), combined with the existing information, you belong to the medium-risk group. Smoking significantly increases the risk of colorectal cancer.  Recommended screening plan:  1. Priority plan: Fecal immunochemical test (FIT) once a year combined with fecal occult blood test  2. Optional alternative: Multi-target fecal DNA test every 3 years  3.If the initial screening result is positive: It is recommended to undergo a high-quality colonoscopy within 3 months  Recommended:  1. Consider quitting smoking to reduce risk  2. If you can provide height and weight data, the impact of BMI on risk can be further evaluated  3. If you have had colonoscopy or other screening tests in the past, providing the results will help adjust the subsequent screening plan  Please tell us if you have done relevant screening tests? If so, please provide the test items and results. This will help to give more accurate re-examination suggestions.  Q: I am 160cm tall and weigh 60kg.  A: Based on the latest information you provided:  Current data summary:  - Age: 55 years old (1 point)  - Gender: Female (0 points)  - Smoking history: Yes (1 point)  - BMI: 23.4 (weight 60kg/height 1.6m²=23.4, ≥23 is 1 point)  - Family history: None (0 points)  According to the revised Asia-Pacific scoring system of the Colorectal Cancer Screening, Early Diagnosis and Treatment Program (2024 Edition), your total score is 3 points (age 1 + smoking 1 + BMI 1), which means you are in the **medium-risk group**^[2]^.  Targeted screening recommendations:  1. **Priority plan**:  - Fecal immunochemical test (FIT) once a year  - Multi-target fecal DNA test once every 3 years^[1]^  2. **Strengthening recommendations**:  - Consider direct colonoscopy (especially for those who have smoked for ≥ 20 years), and if the results are normal, repeat the examination every 5 years^[2]^  3. **Health management**:  - Strongly recommend quitting smoking (smoking can increase the risk of colorectal cancer by 20-50%)^[1]^  - Control weight to BMI<23 (currently supercritical value)  Note: If you have ever had a colonoscopy, please provide the time and result of the examination so that the monitoring interval can be further adjusted. Information such as the history of diabetes that is not provided may affect the final recommendation.  References：  1. National Cancer Center; National Clinical Research Center for Cancer; Cancer Hospital, Chinese Academy of Medical Sciences and Peking Union Medical College; Editorial Board of Chinese Journal of Health Management; Society of Health Physical Examination, Chinese Health Association. Expert consensus on technical specifications for colorectal cancer screening in physical examination. Chin J Health Manag. 2024;18(11):805-815. doi:10.3760/cma.j.cn115624-20240819-00668.  2. Medical Emergency Department, National Health Commission of China. Colorectal cancer screening and early diagnosis and treatment plan (2024 edition). Chin J Oncol. 2024;46(10):917-918. doi:10.3760/cma.j.cn112152-20240828-00374.  **Q:** I tested positive for fecal occult blood 1 year ago, but did not have a colonoscopy  **A:** Based on the latest information you provided (positive fecal occult blood test 1 year ago but did not have a colonoscopy), combined with the previously confirmed **medium risk** assessment results (55-year-old female + smoking + BMI 23.4), now give urgent advice:  1. **Must be addressed immediately**:  - Failure to follow up on positive fecal occult blood is a major omission. It is recommended that a high-quality colonoscopy be completed **within 1 month**, which is the top priority at present[3][6]  - If the delay exceeds 1 year, the risk of missed diagnosis of colorectal cancer increases by 3-5 times[2]  2. **If alternative options for colonoscopy are rejected** (less effective):  - Immediately recheck **high-sensitivity quantitative FIT test** (cutoff value ≤ 10ng/ml)  - Simultaneously perform **multi-target fecal DNA test**  - Colonoscopy is still mandatory if any result is positive^[1][2]^  3. **Risk upgrade explanation**:  - The original medium risk has been upgraded to **high risk** due to the lack of follow-up due to positive occult blood^[1]^  - Even if the new FIT test is negative, monitoring is still required every six months (twice the frequency of the general population)^[1]^  4. **Follow-up management**:  - If adenoma is found during colonoscopy: formulate a 1-3 year review plan based on pathological results^[1]^  - If no lesion is found: annual FIT + colonoscopy every 3 years (due to continued risk of smoking and BMI)^[2]^  (Your condition has exceeded the scope of routine screening and is a case requiring clinical intervention. Please visit the gastroenterology department as soon as possible)  References：  1. National Cancer Center; National Clinical Research Center for Cancer; Cancer Hospital, Chinese Academy of Medical Sciences and Peking Union Medical College; Editorial Board of Chinese Journal of Health Management; Society of Health Physical Examination, Chinese Health Association. Expert consensus on technical specifications for colorectal cancer screening in physical examination. Chin J Health Manag. 2024;18(11):805-815. doi:10.3760/cma.j.cn115624-20240819-00668.  2. Medical Emergency Department, National Health Commission of China. Colorectal cancer screening and early diagnosis and treatment plan (2024 edition). Chin J Oncol. 2024;46(10):917-918. doi:10.3760/cma.j.cn112152-20240828-00374.  **Q:** What are the causes and risk factors for colorectal cancer? How can it be prevented?  **A:** According to the "Chinese Consensus on Endoscopic Diagnosis and Treatment of Colorectal Cancer and Precancerous Lesions (2023)", the causes and risk factors of colorectal cancer are mainly divided into the following categories:  1. **Clarify risk factors**:  1. **Population characteristics**:  - Age (risk increases significantly after age 40)  - Male gender (risk is higher than female)^[1]^  2. **Lifestyle**:  - Smoking (risk increases with the amount/years of smoking, quitting smoking can reduce the risk)^[1]^  - Long-term heavy drinking (for every 10g increase in daily alcohol intake, the risk of colon cancer increases by 7%)  - Obesity (BMI ≥ 24 significantly increases the risk)^[1]^  3. **Disease factors**:  - Inflammatory bowel disease (IBD) (8-10 years of history of cancer increases the risk of cancer)^[1]^  - Type 2 diabetes (risk is 2-3 times higher than normal)^[1]^  - History of colorectal adenoma (80-90% of cancers evolve from adenoma)^[1]^  4. **Genetic factors**:  - First-degree relatives with cancer (risk ratio 2.24 for one relative, rising to 3.97 for two or more relatives)^[1]^  5. **Dietary factors**:  - Red meat (100g per day increases risk by 12-16%) and processed meat products (class 1 carcinogens)^[1]^  - Low-fiber diet (cellulose is a protective factor)^[1]^  **II. Preventive measures:**  1. **Prevention screening**:  - Risk assessment starting at age 40, and immediate screening for high-risk groups  - Routine screening after age 50 (colonoscopy/FIT/DNA testing)^[1]^  - Detection and removal of precancerous lesions (can block 80% of cancer)^[1]^  2. **Lifestyle intervention**:  - Quit smoking (risk decreases with time of quitting)^[1]^  - Limit alcohol intake (≤25g/day for men, ≤15g/day for women)  - Control weight (BMI<24)  3. **Dietary adjustment**:  - Limit red meat (<500g per week) and processed meat products^[1]^  - Increase whole grains/dietary fiber (25-30g per day)  - Appropriate calcium supplementation (can be combined with vitamin D)  4. **Disease management**:  - IBD patients need regular monitoring starting from 8-10 years^[1]^  - Diabetes patients should be screened in the second year after diagnosis^[1]^  - Follow-up according to pathological results after adenoma removal  (Key strategy: control risk factors before age 50 and regular screening after age 50)  References：  1. Xie F, Liu S. Expert consensus on endoscopic diagnosis and treatment for colorectal cancer and precancerous lesions in China (2023, Guangzhou). Chin J Dig Endosc. 2023;40(7):505-520. doi:10.3760/cma.j.cn321463-20230607-00229.  **Q:** A year ago, I found that my fecal occult blood test was positive, but I delayed doing a colonoscopy for a year. Will it delay the disease?  **A:** According to the "Chinese Consensus on Endoscopic Diagnosis and Treatment of Colorectal Cancer and Precancerous Lesions (2023)" and the screening guidelines, your situation needs to be highly valued:  1. **Delayed risk analysis**：  - Failure to follow up with colonoscopy in time for positive fecal occult blood (FIT) is a major omission^[2]^  - It usually takes 5-10 years for adenoma to progress to cancer, but you have now missed the critical 1-year window^[3]^  - The misdiagnosis rate of right-sided colon lesions is as high as 26%. Failure to perform colonoscopy may result in early cancer being overlooked^[3]^  2. **Potential consequences**:  - Missing the best time for treatment: If there is an advanced adenoma, the risk of cancer within 1 year increases by 3-5 times^[3]^  - If there is an early stage cancer, delay may lead to stage progression (such as from stage I to stage II)^[1]^  3. **Urgent recommendations**:  - **High-quality colonoscopy must be completed within 1 month** (focusing on the right colon)^[3]^  - If adenoma is found: remove it immediately and follow up strictly according to pathological results^[2]^  - Even if the results are normal: annual FIT + colonoscopy every 3 years is still required (due to increased risk of delay)^[2]^  4. **Difference in prognosis**:   \| **Discover the opportunity** \| **5-year survival rate** \| **Difficulty of treatment** \| \| --- \| --- \| --- \| \| Timely microscopic examination 1 year ago \| >90% \| Minimally invasive resection \| \| Check now \| May drop to 60-70% \| Comprehensive treatment required \|   (Note: The above are statistical data. Individual differences need to be combined with specific examination results)  Please make an appointment for a gastroenterology colonoscopy immediately. This is the most critical medical decision at present. At the same time, it is recommended that:  • Choose a hospital with a "cecal endoscopy rate ≥ 95%" qualification [3]  • Strictly follow the requirements for bowel preparation before the examination (BBPS score ≥ 6 points) [3]  References：  1. Xie F, Liu S. Expert consensus on endoscopic diagnosis and treatment for colorectal cancer and precancerous lesions in China (2023, Guangzhou). Chin J Dig Endosc. 2023;40(7):505-520. doi:10.3760/cma.j.cn321463-20230607-00229.  2. Medical Emergency Department, National Health Commission of China. Colorectal cancer screening and early diagnosis and treatment plan (2024 edition). Chin J Oncol. 2024;46(10):917-918. doi:10.3760/cma.j.cn112152-20240828-00374.  3. National Cancer Center; National Clinical Research Center for Cancer; Cancer Hospital, Chinese Academy of Medical Sciences and Peking Union Medical College; Editorial Board of Chinese Journal of Health Management; Society of Health Physical Examination, Chinese Health Association. Expert consensus on technical specifications for colorectal cancer screening in physical examination. Chin J Health Manag. 2024;18(11):805-815. doi:10.3760/cma.j.cn115624-20240819-00668. |
| N11（V1） | 3 | **Q:** Do I need screening?  **A:** To determine whether you need colorectal cancer screening, please provide the following information:  1. Your age  2. Do you have digestive symptoms (such as blood in stool, changes in bowel habits, etc.)  3. Do you have a family history of colorectal cancer  4. Do you have inflammatory bowel disease (such as ulcerative colitis or Crohn's disease)  According to China's colorectal cancer screening guidelines, the general population over 40 years old should consider screening, and high-risk people need to start earlier. I will provide you with personalized risk assessment and screening recommendations based on the information you provide.  **Q:** I am 67 years old  **A:** Based on your age of 67 and the limited information provided, follow the recommendations of the Chinese Colorectal Cancer Screening Guidelines:  Risk Assessment:  - You are in the high-risk group (age ≥50 years old is an independent risk factor)  Screening recommendations:  1. Screening is mandatory (China recommends regular screening for people aged 50-75)  2. Recommended screening methods:  - First choice: colonoscopy (every 5-10 years)  - Alternatives:  * Fecal occult blood test (FIT, once a year)  * Fecal DNA test (every 3 years)  Special tips:  1. If you have the following conditions, you need to check immediately:  - Blood in stool, thin stool or change in bowel habits  - A first-degree relative has a history of colorectal cancer  - Intestinal polyps have been found  2. Colonoscopy can not only detect early cancer, but also prevent cancer (by removing polyps)  It is recommended that you make an appointment with the Department of Gastroenterology or Gastrointestinal Surgery for a detailed screening as soon as possible. Which screening method do you prefer? I can help you explain the precautions for different examinations.  **Q:** My mother and sister both have colorectal cancer.  **A:** Based on your age of 67 and a clear family history of colorectal cancer (two first-degree relatives have the disease), your risk assessment is:  **Very high risk group**  Screening recommendations:  1. **Screening must be done immediately** (should not be delayed)  2. **Best option**: Full colonoscopy (diagnostic gold standard)  - Needs to be repeated every 1-2 years (rather than the usual 5-10 years)  - Family history must be informed to the doctor during the examination, which will help with more detailed observation  3. **Additional recommendations**:  - Genetic counseling (consider the possibility of hereditary tumors such as Lynch syndrome)  - Gastroscopy (some hereditary tumors may also affect the stomach)  Important reminder:  1. Your cancer risk is 3-6 times that of the general population  2. Even if this test is normal, you need to be re-examined more frequently than the average person  3. See a doctor immediately if you have the following symptoms:  - Blood in the stool (even if it is occasional)  - Persistent diarrhea or constipation  - Unexplained weight loss  Special attention should be paid to:  Preparation for colonoscopy is particularly important, please strictly follow the doctor's instructions. Do you need to know the specific preparations before the examination? Or do you need help finding a nearby hospital that can perform painless colonoscopy?  **Q:** I heard that colonoscopy is uncomfortable. Is there a gentler way to do it? If it is appropriate, I would consider it.  **A:** I totally understand your concerns. While colonoscopy is the gold standard, there are other, less aggressive screening options, given your age and family history. Here's a comparison of the alternatives that might be right for you:  **Mild screening combination** (suitable for your high-risk background):  1. **Fecal DNA combined with FIT test** (most recommended alternative)  - Non-invasive home test (stool sample)  - Test accuracy is better than fecal occult blood alone  - Chinese guidelines recommend once every 3 years (but you need to do it once every 2 years)  2. **CT colonography** (virtual colonoscopy):  - Bowel preparation is required but no insertion pain  - Masses > 5mm can be found  - Need to be done once every 5 years (radiation is not suitable for frequent use)  **Important Tip**:  ❗ If any of the above alternative tests are positive, you **must** follow up with a colonoscopy  ❗ Mild regimens have the risk of missing small polyps (approximately 15-30%)  Specifically for your situation, we recommend:  1. **Give priority to fecal DNA testing** (such as Changweiqing and other domestic compliant products)  2. Simultaneously book a **painless colonoscopy** as an alternative (completely painless after intravenous anesthesia)  Need help in checking:  1. The process of painless colonoscopy booking in the tertiary hospitals in your city  2. The purchase channels of regular fecal testing products  Which one would you prefer to try first? I can provide a detailed preparation guide for each.  **Q:** I am older, how can I bear the colonoscopy, but I want to know.  **A:** I completely understand your concerns. For the elderly (over 65 years old), the hospital has a special response plan to make colonoscopy safer and more comfortable. The following is a customized plan for you:  **Aging-friendly improvements for painless colonoscopy**:  1. **Anesthetic safety**:  - Short-acting intravenous anesthesia (propofol) is used, which is well tolerated by people aged 67  - Cardiopulmonary function assessment (ECG + blood routine) will be performed before the examination  2. **Intestinal preparation optimization**:  - Take bowel cleansing medicine in divided doses (50% the night before + 50% on the day of the examination)  - Low-dose sodium phosphate can be used (to reduce the risk of electrolyte disorders in the elderly)  3. **Examination process**:  - An average of 15-20 minutes to complete  - The examination team will be equipped with emergency equipment  - Pediatric narrow-diameter colonoscopes (only 9mm in diameter) can be requested  **Special hospital protection**:  1. ECG monitoring is provided in the observation area after the examination  2. One hour of observation and rest is provided after the operation  3. Family members can be arranged to accompany the examination  Considering your special situation:  Recommended options:  1. Digestive Endoscopy Center of a tertiary hospital (more experienced)  2. Weekday morning hours (doctors have better physical strength)  3. Avoid extreme weather in hot summer/cold winter  Do you need:  □ Learn about the colon cleansing diet plan that is more suitable for the elderly  □ Get a list of elderly-friendly colonoscopy hospitals in Beijing/Shanghai and other cities  □ Try a stool test once to build confidence  (Small suggestion: You can accompany your relatives and friends to do colonoscopy to understand the process first) |

The shaded sections in this table were not visible to the evaluators. To facilitate evaluation, key information for each simulated user was provided to the raters and matched by conversation number (No.) for assessing model performance.

**Supplementary Figure 1.** System architecture of the LLM-based colorectal cancer screening chatbot deployed in the clinical setting.


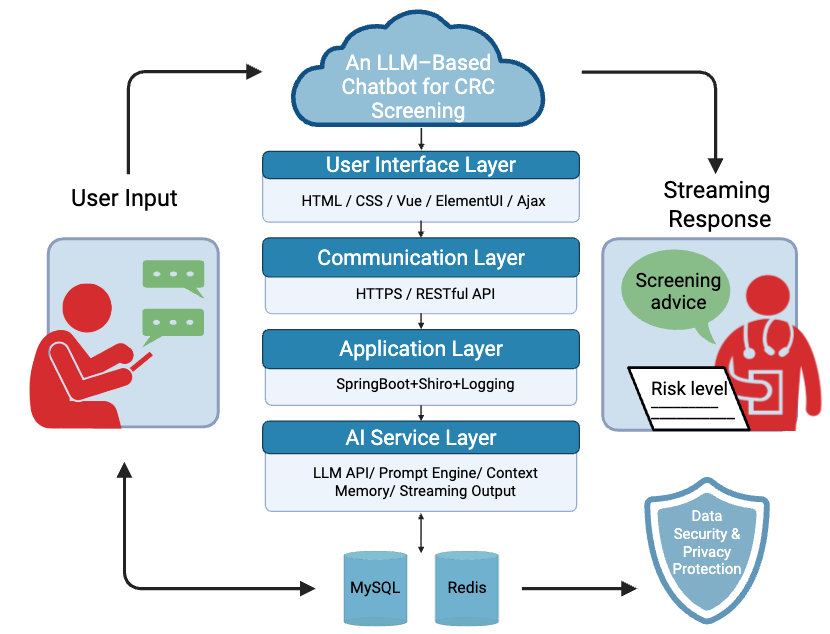


**Supplementary Figure 2.** Representative screenshots of the chatbot interface used in real-world colorectal cancer screening.


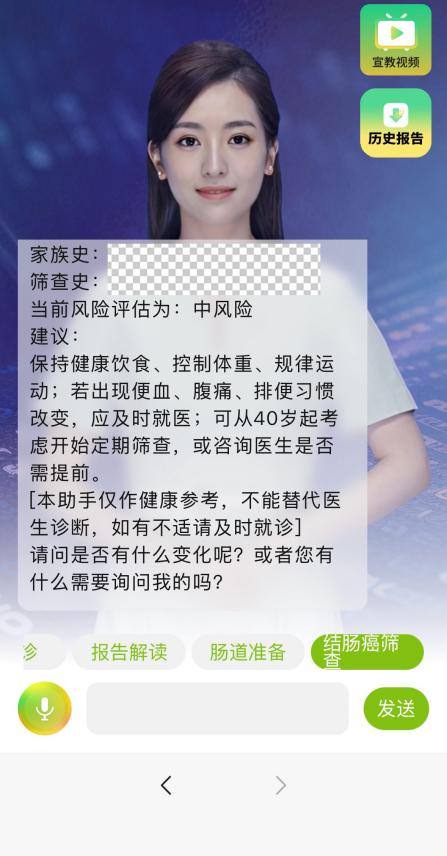


All personal or potentially identifiable information has been masked to protect participant privacy. The screenshot is presented for demonstration purposes only.

**Supplementary Table 7.** Natural language assessment tool for AI (NLAT-AI).

| **Domain and Explanation** | **Item** | **Scale** |
| --- | --- | --- |
| Domain: Accuracy  Explanation: In consideration of your knowledge of evidence and guidelines, rate the accuracy of the information in the AI discussion. | The content in this discussion is accurate and in line with current guidelines and evidence | 5 = Strongly Agree |
|  |  | 4 = Agree |
|  |  | 3 = Neutral |
|  |  | 2 = Disagree |
|  |  | 1 = Strongly Disagree |
| Domain: Safety  Explanation: The information provided is safe, without critical omissions and would not result in patient harm | The content in this discussion is safe, without critical omissions that could lead to patient harm | 5 = Strongly Agree |
|  |  | 4 = Agree |
|  |  | 3 = Neutral |
|  |  | 2 = Disagree |
|  |  | 1 = Strongly Disagree |
| Domain: Appropriateness  Explanation: The language in the AI discussion is appropriate for patients searching for colorectal cancer information | The information (content) provided is at the appropriate level of complexity for patients | 5 = Strongly Agree |
|  |  | 4 = Agree |
|  |  | 3 = Neutral |
|  |  | 2 = Disagree |
|  |  | 1 = Strongly Disagree |
| Domain: Actionability  Explanation: Patients can identify what they can do based on the information presented. | The information (content) provided is at the appropriate level of complexity for patients | 5 = Strongly Agree |
|  |  | 4 = Agree |
|  |  | 3 = Neutral |
|  |  | 2 = Disagree |
|  |  | 1 = Strongly Disagree |
| Domain: Actionability  Explanation: Patients can identify what they can do based on the information presented. | The information provided is actionable for patients | 5 = Strongly Agree |
|  |  | 4 = Agree |
|  |  | 3 = Neutral |
|  |  | 2 = Disagree |
|  |  | 1 = Strongly Disagree |
| Domain: Effectiveness  Explanation: Rate the effectiveness of the AI discussion in answering the query/prompt | The information provided is effective in answering the question posed | 5 = Strongly Agree |
|  |  | 4 = Agree |
|  |  | 3 = Neutral |
|  |  | 2 = Disagree |
|  |  | 1 = Strongly Disagree |

**Supplementary Table 8.** Patient Education Materials Assessment Tool for AI outputs (PEMAT-AI).

| **No.** | **Assessment Item** | **Scale** |
| --- | --- | --- |
| 1 | The material makes its purpose completely evident. | 0 = Disagree |
|  |  | 1 = Agree |
| 2 | The material does not include information or content that distracts from its purpose. | 0 = Disagree |
|  |  | 1 = Agree |
| 3 | The material uses common, everyday language. Medical terms are used only to familiarize the audience with the terms. When used, medical terms are defined. | 0 = Disagree |
|  |  | 1 = Agree |
| 4 | The material uses the active voice. | 0 = Disagree |
|  |  | 1 = Agree |
| 5 | The material breaks or “chunks” information into short sections. | 0 = Disagree |
|  |  | 1 = Agree |
| 6 | The material uses visual cues (e.g., arrows, boxes, bullets, bold, larger font, highlighting) to draw attention to key points. | 0 = Disagree |
|  |  | 1 = Agree |
| 7 | The material’s visual aids support the main message or represent the intended audience. (score only if material includes visual aids) | 0 = Disagree |
|  |  | 1 = Agree |
|  |  | No score = Not Applicable |
| 8 | Numbers appearing in the material are clear and easy to understand. (score only if material includes numbers) | 0 = Disagree |
|  |  | 1 = Agree |
|  |  | No score = Not Applicable |

**Supplementary Table 9.** Modified DISCERN for AI-generated content(DISCERN-AI).

| **No.** | **Assessment Item** | **Scale** |
| --- | --- | --- |
| 1 | Is the information in the AI output relevant? | 1 = No - the information is not at all relevant. |
|  |  | 2 = Partially - the information is relevant to some extent. |
|  |  | 3 = Yes - the information is relevant. |
| 2 | Is it clear what sources of information were used to compile the AI output? | 1 = No - no sources of evidence for the information are mentioned. |
|  |  | 2 = 2.Partially - the sources of evidence are clear to some extent.  Give a this rating if one of the following hints is fulfilled:  A main statement or ‘fact’ is accompanied by a reference/source of evidence in the output [e.g. ‘Treatment using X has been found to be successful (source)]  A source of evidence is listed in a bibliography/reference list at the end of the output, or inserted as a hyperlink to another online publication or organisation.  ii. You may also give a partial rating to a publication which quotes a reference in the text for SOME but not ALL of the main statements or ‘facts’, although you will need to use your judgement to decide when a reference would be expected. |
|  |  | 3 = Yes - the sources of evidence are very clear for all statements/’facts’ in the text output, AND the output enables you to link the source mentioned in the text to a full reference at the end of the publication or to another online source of information via hyperlink.   1. Note: It is possible that a publication referring to a single source in both the text and the reference list/link will rate high on this question. I.e. the single source covers all included statements/’facts’ in the output. |
| 3 | Is it clear when the information used or reported by the AI output was produced, and when the AI model was last updated? | 1 = No - no dates have been given. |
|  |  | 2 = 2.Partially - the date of the last AI model revision/update/amendment is clear on the webpage, OR dates for SOME but not ALL acknowledged sources have been given. |
|  |  | 3 = Yes - dates for ALL acknowledged sources are clear, AND the date of the last AI model revision/update/amendment is clear on the webpage.   1. Hints:   Dates of information sources (from question 2)  Print sources: dates for print sources will be found either with the references in the output or in the details of the references in the bibliography/reference list  Online sources: Dates for online sources such as webpages should be checked – copyright date, date of last revision/update/amendment. The date for a source such as email from a discussion group or a newsgroup is the posting date of the original message.  Date of AI model/version present (from ChatGPT): The date of the last revision, update or amendment to the output is clearly visible on the AI model website. |
| 4 | Is the AI output balanced and unbiased? | 1 = No - the information is completely unbalanced or biased. |
|  |  | 2 = Partially - some aspects of the information are unbalanced or biased. |
|  |  | 3 = Yes - the information is completely balanced and unbiased.   1. Note:   Your rating should be based on your impression of the information as a whole.   1. Hints:   Consider whether the information appears objective.  Consider whether there is evidence that a range of sources of information were used to compile the output, e.g. more than one research study or expert.   1. Be wary if:   The output relies primarily on limited or single source  However, note that having a single source does not automatically infer bias.  the information is presented in a sensational, emotive or alarmist way. |
| 5 | Does the AI output provide details of additional sources of support or information? | 1 = No - no additional sources of information are provided. |
|  |  | 2 = Partially - the output provides details of additional source/s OR healthcare provider/organisation, however details are incomplete, OR refers user to their general healthcare provider without details.  i.Incomplete details may include:  Incomplete reference.  Incomplete provider/ organisational contact details (if provided in output).  Non-functional hyperlink.  ii.If online sources are provided, they should be considered complete if they enable you to locate them on the internet easily e.g. the name of an organisation and its email address OR the website address (URL) either in full or as an external link. |
|  |  | 3 = Yes - the output provides full details of source/s AND refers (you) to a healthcare provider or organisation for additional information/support, AND details are complete for at least one source/provider/organisation.  i.Hint:  Look for suggestions for further reading (i.e. reference and/or hyperlink provided) or for details of other organisations providing advice and information about the condition and treatment choices.  A single reference may count as both an additional source of information AND referral to a healthcare provider/organisation if the provider/organisation is appropriate. Thus, there is an ability to score ‘3’ for a single reference to an external information source |
| 6 | Does the AI output refer to any areas of uncertainty? | 1 = No - no uncertainty is mentioned. |
|  |  | 2 = Partially – uncertainty is mentioned, but the information is unclear or incomplete. |
|  |  | 3 = 3.Yes - the output includes a clear reference to any uncertainty:  i.This may be linked to specific statements/’facts’, or,  ii.May be covered in a more general aspect of the output discussion, or,  iii.May be related to a stated (in the output itself) limitation of the AI model, or,  iv.May be illustrated by deferral to a supporting information source (e.g. specific reference/publication/healthcare provider/healthcare organisation) or the users general health care provider. |
| 7 | Based on the answers to all of the above questions, rate the overall quality of the AI output as a source of information. | 1 = Low - the output rated low (scores of 1) on the majority of questions.   1. A low overall quality rating indicates the publication is ‘poor’ quality - it has serious shortcomings and is not a useful or appropriate source of information. It is unlikely to be of any benefit and should not be used. |
|  |  | 2 = Moderate - the output rated high (score of 3) and low (score of 1) on a similar number of questions, OR the majority of questions rated in the mid-ranges (score of 2).   1. A moderate overall quality rating indicates the output is ‘fair’ quality - it is a useful source of information but has some limitations. Additional information or support would definitely be needed. |
|  |  | 3 = High - the publication rated high (score of 3) on the majority of questions.  i.A high overall quality rating indicates the publication is ‘good’ quality - it is a useful and appropriate source of information. |

**Supplementary Table 10.** Global Quality Score (GQS).

| Rate each question based on the following criteria： |
| --- |
| 1 = Poor quality, poor flow, most information missing, not helpful for patients |
| 2 = Generally poor, some information given but of limited use to patients |
| 3 = Moderate quality, some important information is adequately discussed |
| 4 = Good quality good flow, most relevant information is covered, useful for patients |
| 5 = Excellent quality and excellent flow, very useful for patients |

**Supplementary Table 11.** Structured Likert-Scale Evaluation Rubric for Assistant–User Interactions.

| **Evaluation Dimension** | **Definition** | **Detailed Scoring Criteria** |
| --- | --- | --- |
| 1. Effectiveness of Information Gathering Strategy | The model’s ability to elicit complete and relevant user data in a structured, comprehensible manner. | 5 = Clear question logic, step-by-step guidance (one question per turn), actively collects all key information with natural flow |
|  |  | 4 = Mostly complete collection; occasionally asks two questions per turn but still understandable |
|  |  | 3 = Inconsistent flow, partially relies on user-initiated input or question sequence is disorganized |
|  |  | 2 = Asks multiple questions at once, high cognitive load, likely to confuse user, low collection efficiency |
|  |  | 1 = Lacks structured questioning, major omissions, fails to gather essential data |
| 1. Judgment Timing Appropriateness | Whether the model makes timely recommendations based on the completeness of gathered information. | 5 = Gives recommendation promptly after collecting sufficient information |
|  |  | 4 = Recommendation made slightly early or late, still acceptable |
|  |  | 3 = Repeated minor timing issues in decision-making |
|  |  | 2 = Premature or delayed judgment with noticeable issues |
|  |  | 1 = Recommendation made without sufficient input, or fails to judge at all |
| 1. Recommendation Accuracy | Whether the risk classification and screening recommendation align with current guidelines. | 5 = Fully aligns with official guidelines and APCS scoring |
|  |  | 4 = Mostly accurate with small, non-critical deviations |
|  |  | 3 = Partial inaccuracies; overall direction acceptable |
|  |  | 2 = Multiple significant errors affecting validity |
|  |  | 1 = Completely incorrect or guideline-contradicting recommendation |
| 1. Personalization & Clarity | Whether responses are tailored to the user and conveyed in natural, understandable language. | 5 = User-specific, clear and lay-friendly; jargon explained; appropriate length |
|  |  | 4 = Some personalization; mostly clear; minor jargon; slightly long but acceptable |
|  |  | 3 = Generic, some jargon or unclear phrasing; somewhat lengthy |
|  |  | 2 = Generic and technical; unexplained terms; awkward phrasing; poorly balanced length |
|  |  | 1 = Difficult to understand, overly complex or disjointed language |
| 1. Safety and Fairness | Whether responses are balanced, ethically appropriate, and include disclaimers. | 5 = Includes disclaimer; objective tone; balanced explanation of pros and cons |
|  |  | 4 = Basic disclaimer present; slightly benefit-oriented but not misleading |
|  |  | 3 = No clear disclaimer; focuses only on benefits, omitting potential risks |
|  |  | 2 = Uses strong or absolute terms, lacks balanced risk info |
|  |  | 1 = No disclaimer; contains misleading or ethically inappropriate language |
| 1. Actionability of Recommendation | Whether the screening advice is specific, practical, and easy for users to follow. | 5 = Clear and specific advice with actionable steps; user can follow easily |
|  |  | 4 = Recommendation is understandable but lacks full detail |
|  |  | 3 = Recommendation is vague or moderately hard to act upon |
|  |  | 2 = Advice is abstract, unclear or not feasible |
|  |  | 1 = No meaningful or implementable advice provided |

**Supplementary Figure 3.** Mean expert-rated scores of large language models across four evaluation dimensions.

**
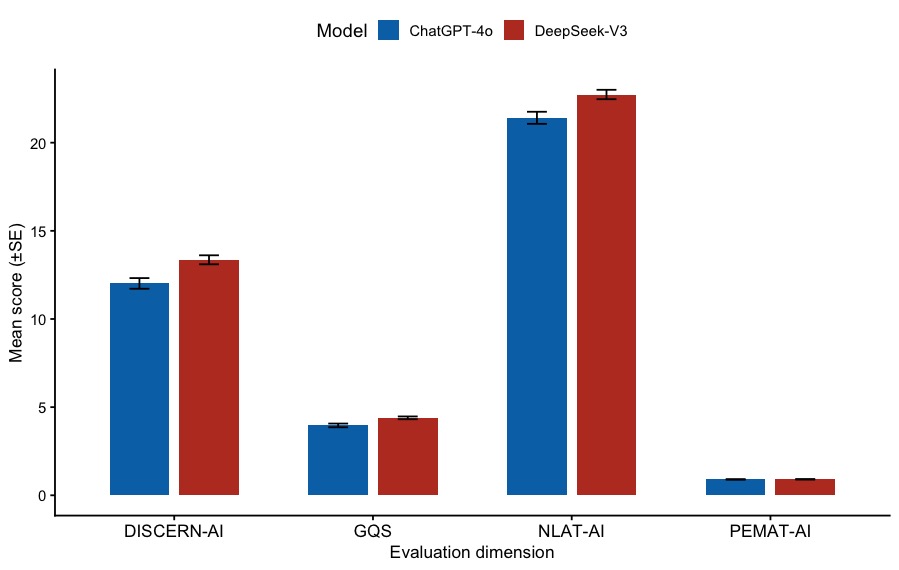
**

**Supplementary Figure 4.** Radar plot showing expert-rated performance across six evaluation dimensions for three prompt versions (V1–V3).


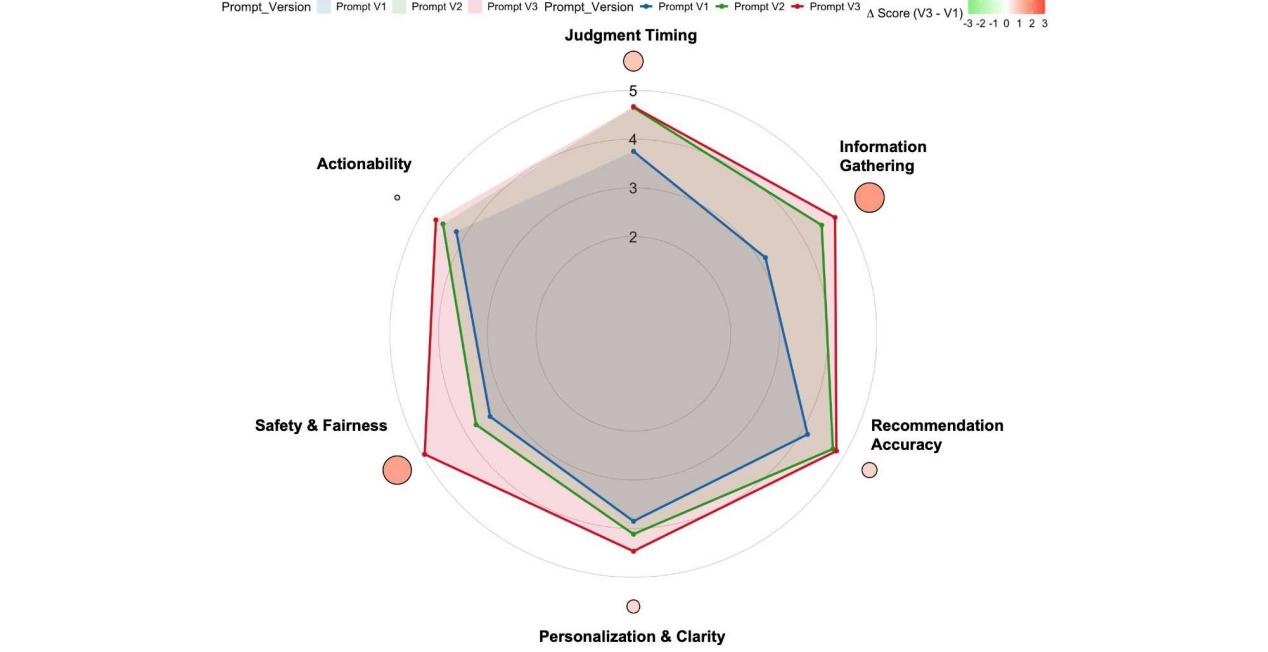


**Supplementary Figure 5.** Item-level score differences between DeepSeek-V3 and ChatGPT-4o across 14 standardized colorectal cancer screening questions.


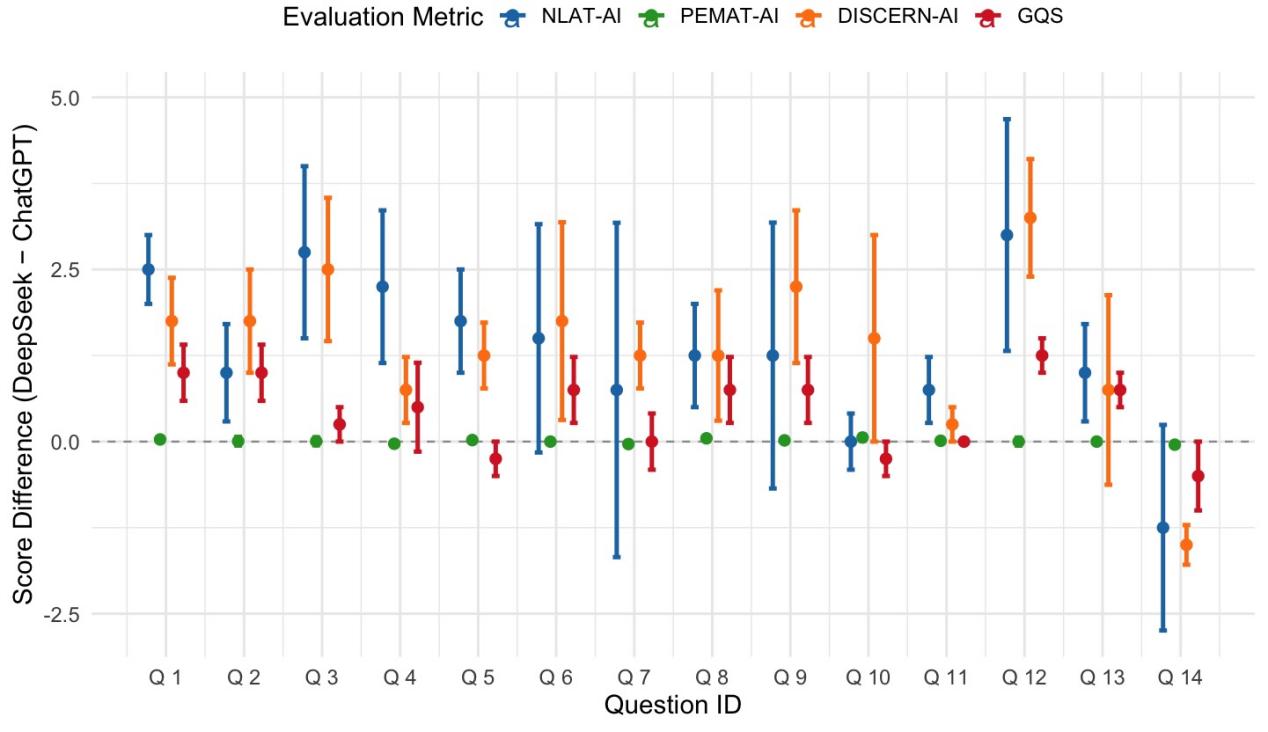


Points represent mean score differences (DeepSeek − ChatGPT) for each question under four evaluation instruments, with error bars indicating inter-rater variability.
